# Genome-wide identification and expression analyses of the *LRR-RLK* gene family in *Actinidia chinensis*

**DOI:** 10.3389/fpls.2025.1577679

**Published:** 2025-09-01

**Authors:** Chang Li, Shengwei Ma, Yulong Tian, Xiaojuan Zheng

**Affiliations:** ^1^ Sanya Nanfan Research Institute, Hainan University, Sanya, China; ^2^ School of Tropical Agriculture and Forestry, Hainan University, Haikou, China; ^3^ Yazhouwan National Laboratory, Sanya, China; ^4^ Wuhan Botanical Garden, Chinese Academy of Sciences, Wuhan, China

**Keywords:** *Actinidia chinensis*, *LRR-RLK*, phylogenetic analyses, *Pseudomonas syringae pv. actinidiae*, abiotic stress response

## Abstract

**Introduction:**

Mihoutao (*Actinidia*) has significant nutritional and economic value, with China leading the world in both cultivation area and production volume. However, bacterial canker caused by Pseudomonas syringae pv. actinidiae (*Psa*) poses a devastating threat to the Mihoutao industry, severely affecting yields. The most effective strategy to combat this disease is through the breeding of resistant varieties. The leucine-rich repeat receptor-like kinase (*LRR-RLK*) family, a major subclass of plant receptor-like kinases, plays crucial roles in plant growth and stress regulation. However, research on *LRR-RLK* genes in Mihoutao remains unexplored.

**Methods:**

In this study, we performed a genome-wide identification of the *LRR-RLK* gene family in *Actinidia chinensis* cv. Hongyang (HY). Their phylogenetic relationships, gene structures, conserved motif, chromosomal location, gene duplication events and promoter cis-elements were analyzed. In addition, we analyzed the genes of the *LRR-RLK* gene family that respond to *Psa* infection based on transcriptomic data and verified their gene expression patterns by qRT-PCR.

**Results and discussion:**

n this study, we identified and manually corrected *LRR-RLK* genes in two haplotypes of HY, resulting in a nonredundant set of 394 *AcLRR-RLK* genes. Phylogenetic analysis revealed that these *AcLRR-RLK* genes are classified into 21 subfamilies distributed across 29 chromosomes. Gene structure analysis revealed high diversity in the LRR domains of *AcLRR-RLK* genes, while the kinase domains were relatively conserved. Analysis of cis-acting elements further suggested the involvement of *AcLRR-RLK* genes in critical biological processes such as the light response, hormone response, growth and development, and biotic and abiotic stresses in HY. Furthermore, transcriptomic analysis across different tissues revealed that the majority of AcLRR-RLK genes play a role in five tissues: root, stem, leaf, flower, and fruit. Additionally, transcriptomic analysis under Psa infection indicated that, HY activated its defense response, with an increasing number of *AcLRR-RLK* genes responding to pathogen invasion. Among these genes, 48 *AcLRR-RLK* genes were identified as core genes responsive to *Psa*, and genes from subgroup XII may play a critical role in the defense mechanism against pathogen invasion. Our study provides an in-depth investigation into the characteristics and functions of the *AcLRR-RLK* gene family in A. chinensis, laying a molecular foundation for further disease-resistant and high quality breeding efforts.

## 1 Introduction

Pattern recognition receptors (PRRs) identify conserved pathogen-associated molecular patterns (PAMPs) and activate pattern-triggered immunity (PTI) to restrict the pathogenicity of pathogenic bacteria. Receptor-like kinases (RLKs) are among the primary PRRs in plants (Ngou et al., 2022b). RLKs are widely present in plants, among which leucine-rich repeat receptor-like kinases (*LRR-RLK*) constitute the largest subfamily within the plant receptor-like kinase family (Shiu and Bleecker, 2001). Phylogenetic studies conducted on 33 plant species have revealed that the average number of *LRR-RLK* genes in angiosperms is approximately 250 per species (Dufayard et al., 2017; Ngou et al., 2022a). The *LRR-RLK* proteins consist of three typical domains, including an extracellular domain (ECD), a single transmembrane domain (TM), and an intracellular kinase domain (KD). The extracellular LRR domain is responsible for perceiving external signals, whereas the intracellular kinase domain activates kinase activity through autophosphorylation, which subsequently phosphorylates specific substrates to activate downstream signaling (Gou et al., 2010). The kinase domain is a conserved domain within the RLK gene family; therefore, the classification of RLKs relies primarily on phylogenetic analysis based on the kinase domain. The plant RLK family can be subdivided into 60 subfamilies on the basis of kinase phylogeny, with *LRR-RLK* genes further classified into 21 subfamilies. These subfamilies are named according to the subfamily classification of *Arabidopsis thaliana LRR-RLK* genes, with Roman numerals used for naming (Shiu et al., 2004). In addition, some *LRR-RLK* genes contain atypical domains, such as TM_EphA1 and AsmA_2 in the LRR-II, LRR-XI-1, and LRR-Xb subfamilies of *Phaseolus vulgaris* L. (da Silva Dambroz et al., 2023).

*LRR-RLK* genes play crucial roles in plant defense against pathogens, with the XII subgroup containing numerous genes involved in pathogen recognition, including *FLS2*, *EFR*, and *Xa21* (Dufayard et al., 2017; Liu et al., 2017). *LRR-RLK* genes are primarily responsible for recognizing PAMPs composed of proteins and peptides, such as bacterial flagellin and transcription elongation factors (Chinchilla et al., 2006; Zipfel et al., 2006). For example, in the model plant *Arabidopsis thaliana*, *FLS2* senses 22 amino acid residues at the N-terminus of flagellin (flg22) and subsequently forms a heterodimer with the *BAK1* protein, initiating the PTI response (Chinchilla et al., 2006). *LRR-RLK* genes also participate in responses to abiotic stresses such as drought and salinity. In maize, the overexpression of *SbER2-1* enhances water use efficiency and the net photosynthetic rate under drought stress, indicating a significant role of *SbER2-1* in the plant drought stress response (Li et al., 2019). Additionally, some *LRR-RLK* genes are involved in regulating plant growth and development as well as hormone signaling pathways. For example, *XIP1/CEPR1* and *CEPR2* in *Arabidopsis thaliana* regulate both lateral root primordium initiation and local lateral root formation (Dimitrov and Tax, 2018).

*Actinidia* (commonly known as Mihoutao in Chinese) is a deciduous climbing fruit tree belonging to the *Actinidiaceae* family and is native to China, where it has abundant wild resources and a wide geographical distribution. In this paper, we use the name Mihoutao to refer to *A. chinensis*, reflecting its Chinese origin. The cultivation area of this industry has been expanding worldwide since the domestication of Mihoutao in the 20th century. According to the latest statistics from the United Nations Food and Agriculture Organization (FAO) in 2024 (https://www.fao.org/statistics/en/), China ranks first in the world in both the area and production of Mihoutao, accounting for 69.58% and 52.44% of the world’s total harvested area and production, respectively. The pathogenic bacterium of bacterial canker in Mihoutao (*A. chinensis*) is *Pseudomonas syringae* pv. *actinidiae* (*Psa*), a devastating disease that threatens Mihoutao production (Chapman et al., 2012). Currently, cultivating resistant varieties is the most effective way to prevent and control major Mihoutao diseases.

Owing to the importance of *LRR-RLK* genes in plant growth and development, as well as in response to biotic and abiotic stresses, current studies have identified and characterized the *LRR-RLK* gene family in various plant species, including *Oryza sativa* (Sun and Wang, 2011), *Arabidopsis thaliana* (Man et al., 2020), *Saccharum* (Ding et al., 2024), *Arachis hypogaea* L (Wang et al., 2024)., and *Liriodendron chinense* (Mu et al., 2024), among others. However, it has been shown that the *LRR-RLK* gene family has significant species specificity. For example, the gene expansion patterns of *Arabidopsis* and rice differ significantly (Shiu et al., 2004), the LRR-I subfamily of *Cruciferae* and mosses is specifically amplified (Kileeg et al., 2023), and the structure and expression patterns of some subfamilies in sugarcane and peanut showed uniqueness (Ding et al., 2024; Wang et al., 2024). More critically, there is a specialized mutualistic relationship between Mihoutao and the pathogen *Psa*, a Mihoutao-specific pathogen whose infection reprograms the gene expression profile. Therefore, it is necessary to analyze and characterize the Mihoutao *LRR-RLK* gene family. The genome of *A. chinensis* Hongyang, has been assembled at the haplotype level (Yue et al., 2023), but *LRR-RLK* family analysis and comparisons at the haplotype level have not been reported. In this study, we identified and classified *LRR-RLK* genes separately from the HapA and HapP genomes. Additionally, via the use of multiple transcriptomic datasets collected from public databases (Song et al., 2019), we conducted a comprehensive analysis of the expression patterns of the *LRR-RLK* genes in the roots, stems, leaves, flowers, fruits, and under disease stress conditions. Through this analysis, we explored *LRR-RLK* genes related to Mihoutao growth, development, and disease resistance, laying a solid foundation for future functional study.

## 2 Materials and methods

### 2.1 Annotation and physicochemical properties of the *LRR-RLK* genes in *A. chinensis*

The protein sequences of the two haplotypes of Hongyang (Yue et al., 2023) were annotated for *LRR-RLK* genes. Conserved kinase domains (PFAM PF00069.26 and PF07714.21) were searched in the filtered protein sequences using HMMER v3.4. Sequences containing kinase domains were annotated for transmembrane and LRR domains using TMHMM v2.0 and InterProScan v5.52 (Sonnhammer et al., 1998; Zdobnov and Apweiler, 2001). Protein sequences containing LRR, TM, and kinase domains were identified as *LRR-RLK* genes. The annotated *LRR-RLK* genes were manually curated using IGV-GSAman (Robinson et al., 2023) to correct any gene structure errors arising from inaccurate genomic annotations. *LRR-RLK* gene structures were manually corrected using transcriptome data. We performed comparative analysis of genome-annotated *LRR-RLK* gene structures with transcriptome-derived structures from RNA-seq alignments. Consistent structures were validated, while discrepancies underwent manual correction based on transcriptomic evidence, followed by structural domain re-annotation for validation. Genes lacking transcriptomic support retained original annotations. The genes that underwent manual curation with IGV-GSAman were renamed accordingly, such as Achv4a03GSAman00001.t1. Among the 394 genes, 20 *AcLRR-RLK* genes were manually corrected based on RNA-seq evidence. The protein sequences of *LRR-RLK* genes from different subfamilies in *Arabidopsis thaliana* were downloaded from the TAIR website (https://www.arabidopsis.org/). The ExPASy ProtParam tool (Wilkins et al., 1999) (http://web.ExPASy.org/protparam/) was used to predict the physicochemical parameters of the proteins. WoLF PSORT (Horton et al., 2007) (https://wolfpsort.hgc.jp/) was employed to generate subcellular localization predictions.

### 2.2 Phylogenetic and conserved motif analysis of the *AcLRR-RLK* genes

The kinase sequences were extracted from the *AcLRR-RLK* sequences and merged with the kinase sequences of *Arabidopsis thaliana LRR-RLK*. Multiple sequence alignment was performed using MAFFT software (Katoh et al., 2002). The aligned sequences were then utilized by Fasttree software (Price et al., 2010) to construct a phylogenetic tree via maximum likelihood with 500 bootstraps. The phylogenetic tree was visualized and polished through iTOL (Letunic and Bork, 2021) (https://itol.embl.de/).

### 2.3 Gene structure and conserved motif identification

The protein sequences of *AcLRR-RLK* genes were analyzed using the MEME suite (Bailey et al., 2009) (http://memesuite) with the following parameters: optimum width, 5-60; several repetitions; and maximum number of motifs, 15, to determine conserved motifs. InterProScan v5.52 software (Zdobnov and Apweiler, 2001) was used to identify the domains containing conserved motifs. To illustrate the structure of the *AcLRR-RLK* gene, TBtools (Chen et al., 2023) was employed to generate the exon–intron arrangement of each *AcLRR-RLK* based on the gff file of *A. chinensis*.

### 2.4 Synteny, gene duplication and gene chromosomal location analysis

On the basis of the genome annotation of the two haplotypes of Hongyang, chromosomal location information for the *AcLRR-RLK* gene was obtained. BLAST (Camacho et al., 2009) software and JCVI (Tang et al., 2024) software were used for synteny analysis. To visualize the results, TBtools software was used to locate the *LRR-RLK* genes on the corresponding chromosomes and illustrate the gene collinearity within each haplotype.

### 2.5 Cis-acting regulatory element analysis of the *AcLRR-RLK* genes

The 2000 bp promoter sequence upstream of the start codon of the *AcLRR-RLK* genes was extracted for further analysis. The cis-acting elements were predicted using the PlantCare website (http://bioinformatics.psb.ugent.be/webtools/plantcare/html/) (Lescot et al., 2002). After summarizing and counting different types of cis-acting elements, visualization was performed using the R package ggplot2 and TBtools software.

### 2.6 Gene expression analysis of the *AcLRR−RLK* family

Transcriptomic data from different tissues of Hongyang (PRJNA888809) and from various time points after infection with *Psa* (PRJNA514180) were collected from NCBI. The transcriptomic data were filtered using the fastp software (Chen et al., 2018). The expression levels of the nonredundant Hongyang genomic cDNA sequences were calculated using the filtered transcriptomic data and salmon software (Patro et al., 2017; Song et al., 2019). To identify expressed genes and minimize background noise, we applied a TPM (Transcripts Per Million) > 1 threshold, consistent with common practice in transcriptomic analyses (Hebenstreit et al., 2011; Hart et al., 2013). The TPM values were normalized via min–max normalization: Xnorm = (X - Xmin)/(Xmax - Xmin), which scales the expression values to a range between 0 and 1. This normalization was used for heatmap visualization only. For statistical testing, raw TPM values were used in edgeR. Three biological replicates per tissue/time point were analyzed. The expression patterns of *AcLRR-RLK* genes in different tissues were visualized using TBtools. Differentially expressed genes at various time points post-infection were analyzed using the edgeR package in R. Genes with unadjusted p-value < 0.05 and fold-change > 1.5 were considered significantly differentially expressed. Differentially expressed genes were further visualized as volcano plots and heatmaps using the R package ggplot and pheatmap software.

### 2.7 qRT-PCR validation of the *AcLRR−RLK* genes

The *A. chinensis* cv. Hongyang was used for quantitative real-time PCR (qRT-PCR) analysis in this study. One-week-old leaves of cv. Hongyang plants were selected for *Psa* inoculation and water treatment. On the day of inoculation, a fresh bacterial suspension of *Psa* at a concentration of 1×10^6^CFU/mL was prepared using sterile water containing 0.01% Tween-20. The abaxial surface of each leaf was gently inoculated with a brush soaked in the bacterial suspension. Six plants were inoculated per treatment group (*Psa*), and mock-inoculated plants were treated with sterile water containing 0.01% Tween-20 only.

Seven representative *AcLRR-RLK* genes were selected for qRT-PCR validation. At 0, 12, 24, 48, and 96 hours after inoculation (hai), three containers from each treatment were randomly selected for sampling. Each biological replicate consisted of pooled leaf samples from three plants per container. All samples were immediately flash-frozen in liquid nitrogen and stored at −80°C until RNA extraction. Total RNA was extracted using the Eastep Super Total RNA Extraction Kit (Shanghai Promega, LS1040). Single-stranded cDNA was synthesized using HiScript III 1st Strand cDNA Synthesis Kit (+gDNA wiper) (Vazyme, R312). Reverse transcription quantitative real-time polymerase chain reactions (qRT-PCR) were performed on HiScript II One Step qRT-PCR SYBR Green Kit (Vazyme, Q221). The qRT-PCR reaction mixture consisted of 10 µL SYBR Green, 0.4 µL of each primer, 6.2 µL ddH_2_O, and 3 µL cDNA, totaling 20 µL. The qRT–PCR reaction conditions were as follows: 30s of pre-denaturation at 95°C, followed by 40 cycles of denaturation at 95°C for 10 s and annealing at 60°C for 30 s. A melting curve analysis was performed immediately after amplification, consisting of denaturation at 95°C for 15 s, annealing at 60°C for 60 s, and a final denaturation at 95°C for 15 s to confirm the specificity of amplification products. Each target gene and sample were set up with 3 technical replicates and 2 biological replicates. The actinidia Actin gene served as the internal reference (Bai, 2023), and primers were designed using Primer 5.0 software (**Supplementary Table S7**). Relative expression levels were calculated using the 2⁻ΔΔCt method. Data significance analysis was performed using SPSS 27.0 software, and the expression results were visualized using the R package ggplot2.

## 3 Results

### 3.1 Identification and manual correction of the *A. chinensis LRR-RLK*

On the basis of the typical domains of *LRR-RLK* genes (LRR, TM and kinase domains), the protein sequences of the HapA and HapP genomes of *A. chinensis* were annotated, and the longest sequence for each transcript of the same gene was selected. To ensure the accuracy of the gene structures, IGV-GSAman software was used to manually correct each *LRR-RLK* gene on the basis of transcriptomic data and domains. After manual correction, the gene names of the *LRR-RLK* genes were renamed, and a total of 336 and 351 *AcLRR-RLK* protein sequences were ultimately retained in the HapA and HapP genomes, respectively (**Supplementary Table S1**). Through collinearity analysis, the *AcLRR-RLK* genes that have a collinear relationship between HapA and HapP were identified, and on the basis of this collinear relationship, redundant *AcLRR-RLK* genes (394 genes) were screened for further analysis (**Supplementary Table S1**). Structural annotation revealed that 70% of *AcLRR-RLK* genes started with the LRRNT_2 domain at the N-terminus and that nearly all ended with the Pkinase/PK_Tyr_Ser-Thr domain at the C-terminus. Some *AcLRR-RLK* genes, in addition to the typical domains, also included various types of atypical domains (**Supplementary Figure S1**). In *A. chinensis*, 154 (39%) *AcLRR-RLK* genes contained atypical domains (**Supplementary Table S1**; **Supplementary Figure S2**). The most common atypical domains were TM_ErbB1 and Syndecan (**Supplementary Figure S2**). Nine genes carried the TM_ErbB1 domain, and 11 genes carried the Syndecan domain. Syndecan is a multifunctional transmembrane heparan sulfate cell surface receptor that may play a role in early development in both plants and animals (Rawson et al., 2005; Rhiner et al., 2005; De Rossi and Whiteford, 2013). TM_ErbB1 belongs to the epidermal growth factor receptor (ErbB) family, a class of receptor tyrosine kinases (RTKs) that play key roles in cell growth, proliferation, and differentiation (Bocharov et al., 2008; Mineev et al., 2010; Bocharov et al., 2012; Endres et al., 2013; Bragin et al., 2016). These results suggest that there are no significant differences in the number or domain types of *AcLRR-RLK* genes between the two haplotypes. The atypical domains carried by *AcLRR-RLK* genes are associated primarily with the growth and development of *A. chinensis*. These results suggest that *AcLRR-RLK* genes that contain TM_ErbB1 and Syndecan atypical domains may be involved in the growth and development of *Actinidia chinensis* cv. Hongyang.

### 3.2 Physicochemical properties of proteins in *A. chinensis*

The physicochemical properties of the *AcLRR-RLK* proteins were analyzed via the ExPASy ProtParam tool (Wilkins et al., 1999) (**Supplementary Table S1**). The protein sequence length of *AcLRR-RLK* genes varies considerably. The amino acid length is in the range of 255-1646. The molecular weight varies from 27.86 kDa to 178.46 kDa. The proteins exhibited varying levels of hydrophilicity, ranging from -0.42 to 0.249. According to established criteria, a pI less than 7 indicates an acidic protein, and an instability index less than 40 indicates a stable protein. In *A. chinensis*, the predicted theoretical pI of the proteins was in the range of 4.85–9.73. Among them, 131 *AcLRR-RLK* genes had a theoretical pI greater than 7, making them positively charged in acidic solutions. The protein instability index was in the range of 24.29–56.66. Among the 394 *AcLRR-RLK* genes, 208 were stable acidic proteins, 80 were stable basic proteins, 55 were unstable acidic proteins, and 51 were unstable basic proteins.

Furthermore, according to the subcellular localization prediction of WoLF PSORT, 48% of *AcLRR-RLK* proteins in *A. chinensis* are present on the plasma membrane (**Supplementary Table S1**). However, only one *AcLRR-RLK* is located in the peroxisome.

### 3.3 Phylogenetic analyses of the *AcLRR-RLK* gene family

**Figure 1:**
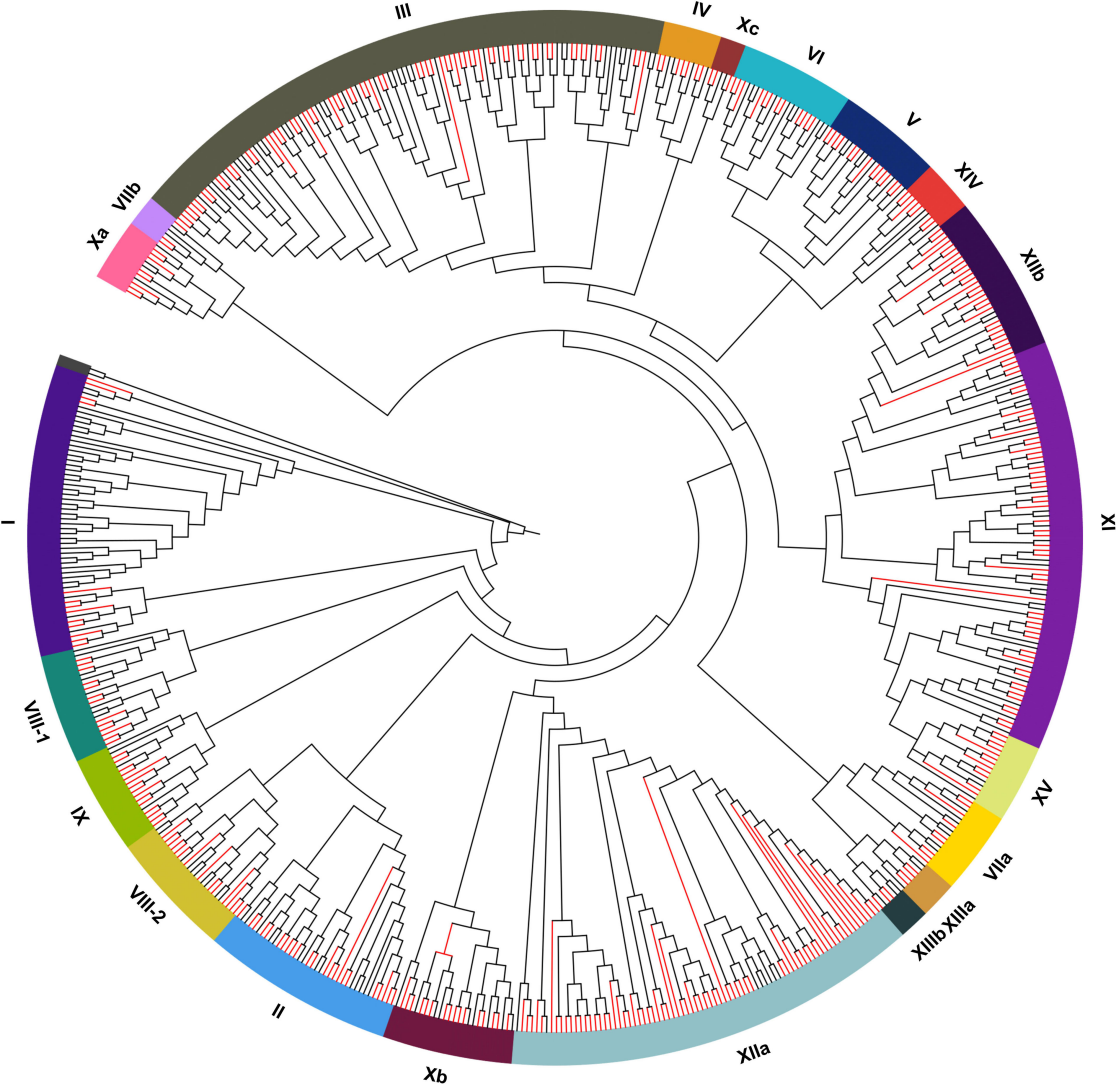
Phylogenetic trees of *LRR-RLK* genes in *A. thaliana* and *A. chinensis*. The red branch represents the *LRR-RLK* genes in *A. chinensis*. The black branch represents the *LRR-RLK* genes in *A. thaliana*.

The phylogenetic relationship of the *AcLRR-RLK* gene family was analyzed on the basis of the classification of the model plant *Arabidopsis thaliana*. The phylogenetic tree was constructed using multiple sequence alignment results from the kinase domain. The results indicate that *AcLRR-RLK* genes can be divided into 21 subfamilies (**Figure 1**; **Supplementary Table S1**), which is consistent with the findings in *Arabidopsis thaliana*, and named using Roman numerals on the basis of a published report (Shiu et al., 2004). Among the *AcLRR-RLK* genes in *A. chinensis*, Group XIIa and III was the largest subfamily, including 72 and 65 *AcLRR-RLK* genes, respectively. The *LRR-RLK* XIIa subfamily, which commonly plays a central role in pathogen recognition and immune response, shows significant gene expansion in many plants (Magalhães et al., 2016). The expansion of XIIa is likely to be driven primarily by coevolutionary selection pressures in a host-pathogen “arms race”. But group III also occupies a high proportion in rice (10.7%) and *Arabidopsis* (19.6%) (Shiu et al., 2004). It suggests that III is generally conserved in dicotyledons and may be involved in core functions such as organ morphogenesis and hormone signaling. This is followed by groups XI, which contain 54 genes. The remaining groups had no more than 30 *AcLRR-RLK* genes. The smallest group is Xc, containing only 3 *AcLRR-RLK* genes. Similarly, Xc is also extremely underrepresented in other plant species (0.8% in *Arabidopsis*, 0.3% in rice) (Shiu et al., 2004), suggesting that it is functionally highly conserved and likely restricted to the underlying signaling pathway, and that natural selection has not driven its expansion. From the perspective of domains, the extracellular domains of groups I and VIII-2 contain not only the LRR domain but also the Malectin domain (**Supplementary Figure S1**). In group I, the Malectin domain precedes the LRR domain, whereas in group VIII-2, it follows the LRR domain. These findings suggest that the largest group of *AcLRR-RLK* genes is group III, whereas the smallest groups are groups VIIb and XIIIb. Additionally, groups I and VIII-2 possess both Malectin and LRR domains in their extracellular domains.

### 3.4 Conserved motif gene structure and domain conservation analyses of *AcLRR-RLK* genes in *A. chinensis* HapA and HapP

**Figure 2:**
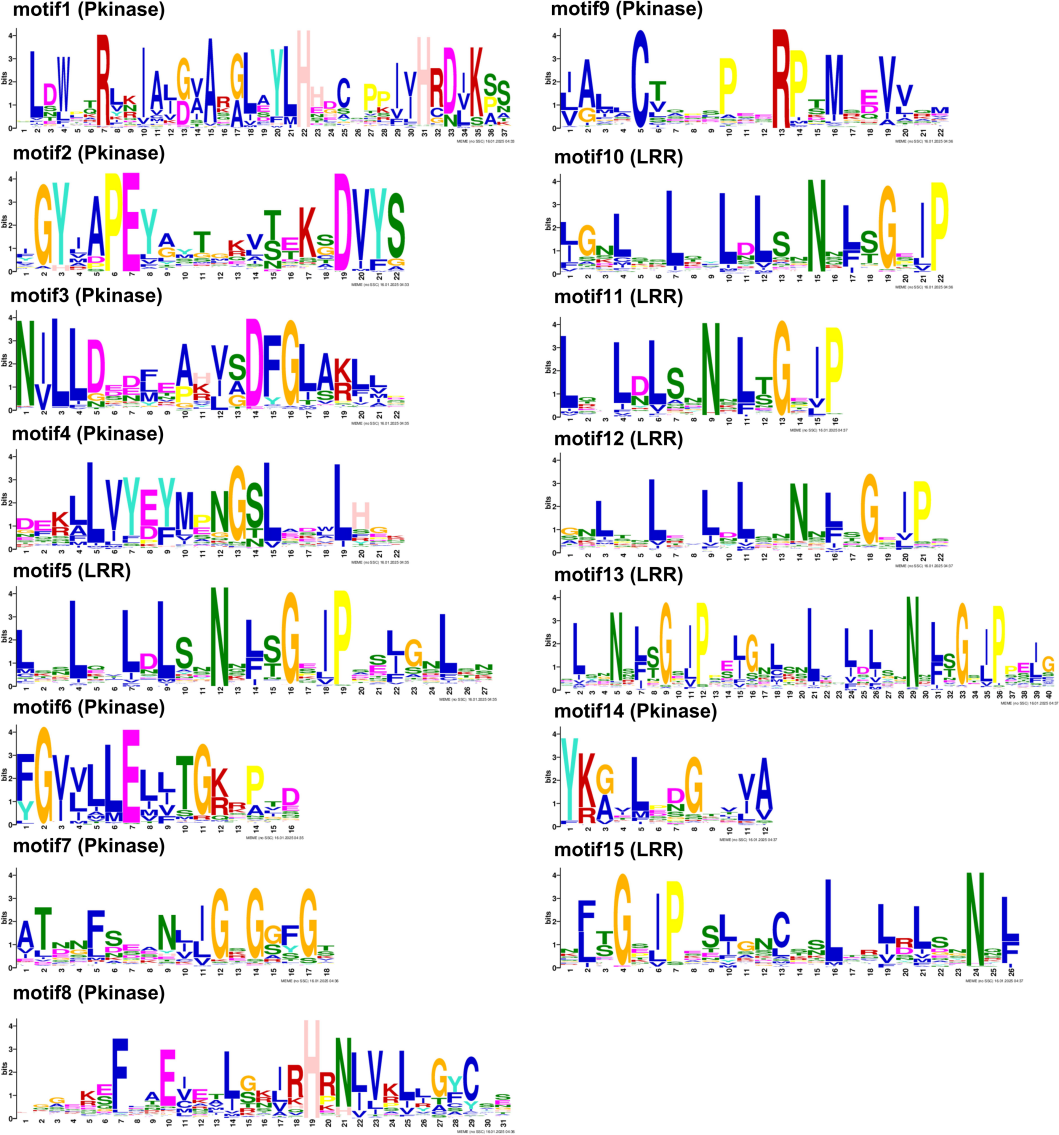
Conserved motifs of *AcLRR-RLK* genes in *A. chinensis*. If the bit value of the amino acid at this position is less than 1, it is represented by x; for 2 > bits ≥ 1, it is represented by a lowercase letter; for 3 > bits ≥ 2, it is represented by a capital letter; and for bits ≥ 3, it is represented by a bold capital letter. The text in parentheses indicates the domain in which the motif resides.

Conserved motif analysis of *AcLRR-RLK* genes was performed using the MEME suite, resulting in the identification of 15 motifs (**Figure 2**). In *AcLRR-RLK* genes, nine conserved motifs were identified in the kinase domain, including motif 1, motif 2, motif 3, motif 4, motif 6, motif 7, motif 8, motif 9, and motif 14, while the remaining motifs were located in the LRR domain. Notably, motif 6 in the kinase region was present in 97.5% of *AcLRR-RLK* genes. Motif 5, motif 10, motif 11 and motif 12 in the LRR region were present in more than 95% of the *AcLRR-RLK* genes. Further observation revealed that some conserved motifs in the LRR region were similar, such as motif 5, motif 10 and motif 11, whereas the conserved motifs in the kinase region were quite different. These results indicate that the kinase domain of *AcLRR-RLK* genes is highly conserved and that the conserved motifs differ greatly. Although the overall diversity of LRR regions is high, some conserved fragments and conserved regions still have certain repeatability. These results are consistent with reported studies (Kileeg et al., 2023). It is further confirmed that the diversity of LRR domains among subfamilies drives the functional diversification of the *LRR-RLK* genes, whereas the kinase domains are highly conserved within the family to maintain the core function of signal transduction.

**Figure 3:**
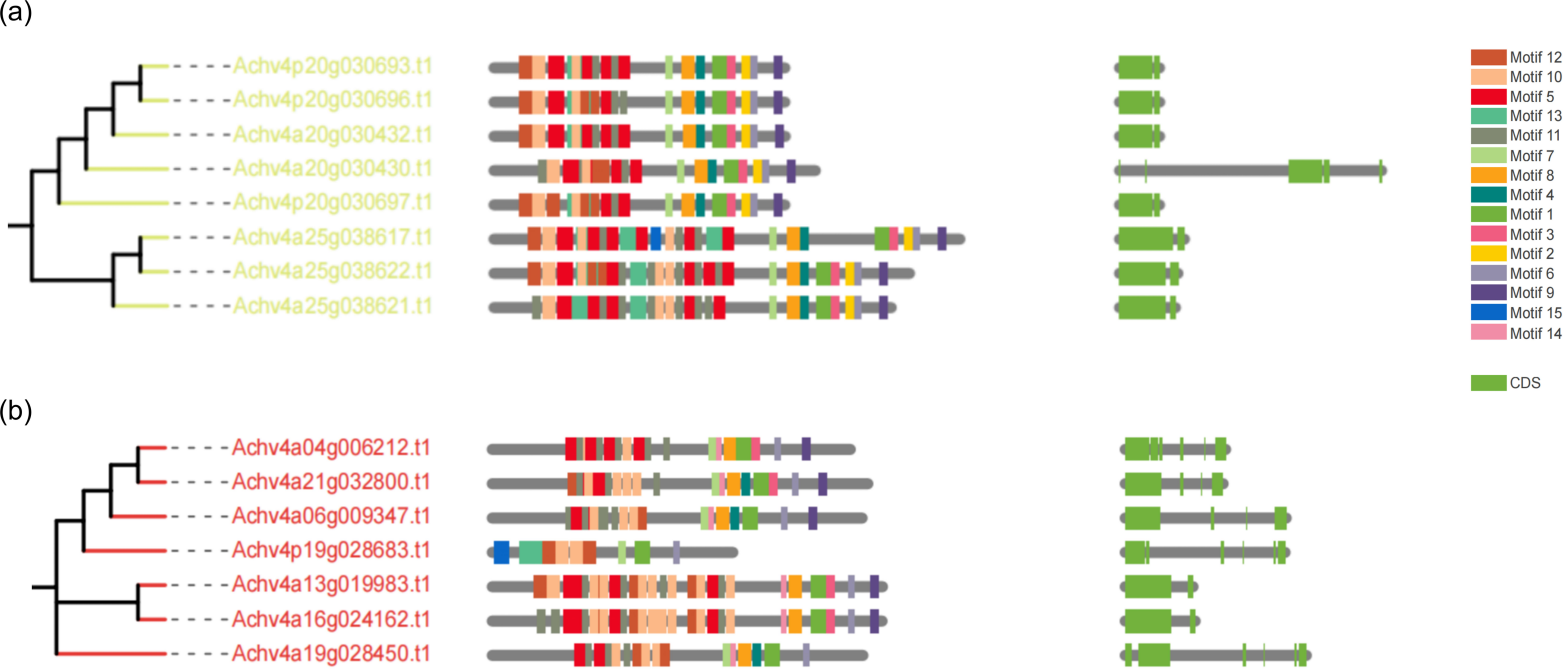
Phylogenetic tree, conserved motifs, and gene structure of group XV and XIV *AcLRR-RLK* genes in *A*. *chinensis*. **(a)** Phylogenetic tree, conserved motifs, and gene structure of group XV *AcLRR-RLK* genes in *A*. *chinensis*. **(b)** Phylogenetic tree, conserved motifs, and gene structure of group XIV *AcLRR-RLK* genes in *A*. *chinensis*. The *AcLRR-RLK* phylogenetic tree was constructed on the basis of kinase sequences. The arrangement of conserved motifs in each *AcLRR-RLK* was visualized using conserved motif information, with colored boxes representing motifs 1–15. The *AcLRR-RLK* gene structure in the cds region was visualized, with the green boxes representing the cds regions.

We further investigated the conserved motif arrangement and gene structure distributions across different groups (**Supplementary File 1**). From the perspective of kinase domains, all subfamilies of *AcLRR-RLK* genes contain motifs 1, 3, 4, 6, 7, 8, and 9, and within the same subfamily, the arrangement of these motifs on the genes is similar. Interestingly, motif 14 is present in all subfamilies except for XV (**Figure 3a**), and motif 2 is present in all subfamilies except for XIV (**Figure 3b**). In terms of the LRR domain, the number of conserved motifs carried by different *AcLRR-RLK* subfamilies is in the range of 1–42. Some larger subfamilies, such as XIIa and XIIb, show significant variation in the number of conserved motifs in the LRR region among their members (**Supplementary File 1**). Overall, among the 21 subfamilies, members of subfamilies V and I generally carry the fewest conserved motifs, whereas members of subfamily XI carry the most conserved motifs. The number of conserved motifs carried by members within the XIIa and XIIb subfamilies varies significantly, which may reflect greater genetic variation within these two subfamilies. In terms of gene structure, most subfamilies have 2-4 exons, with subfamilies I, II, V, VI, VIII-1, VIII-2, and XIIIb typically having more than 10 exons. In *A. chinensis*, subfamilies with more exons tend to have elongated exons and larger intron structures. Within the same subfamily, the motif carrying patterns, sequence arrangement, and gene structure of the members are relatively conserved.

### 3.5 Analysis of cis-regulatory element distribution in *AcLRR-RLK* promoters

**Figure 4:**
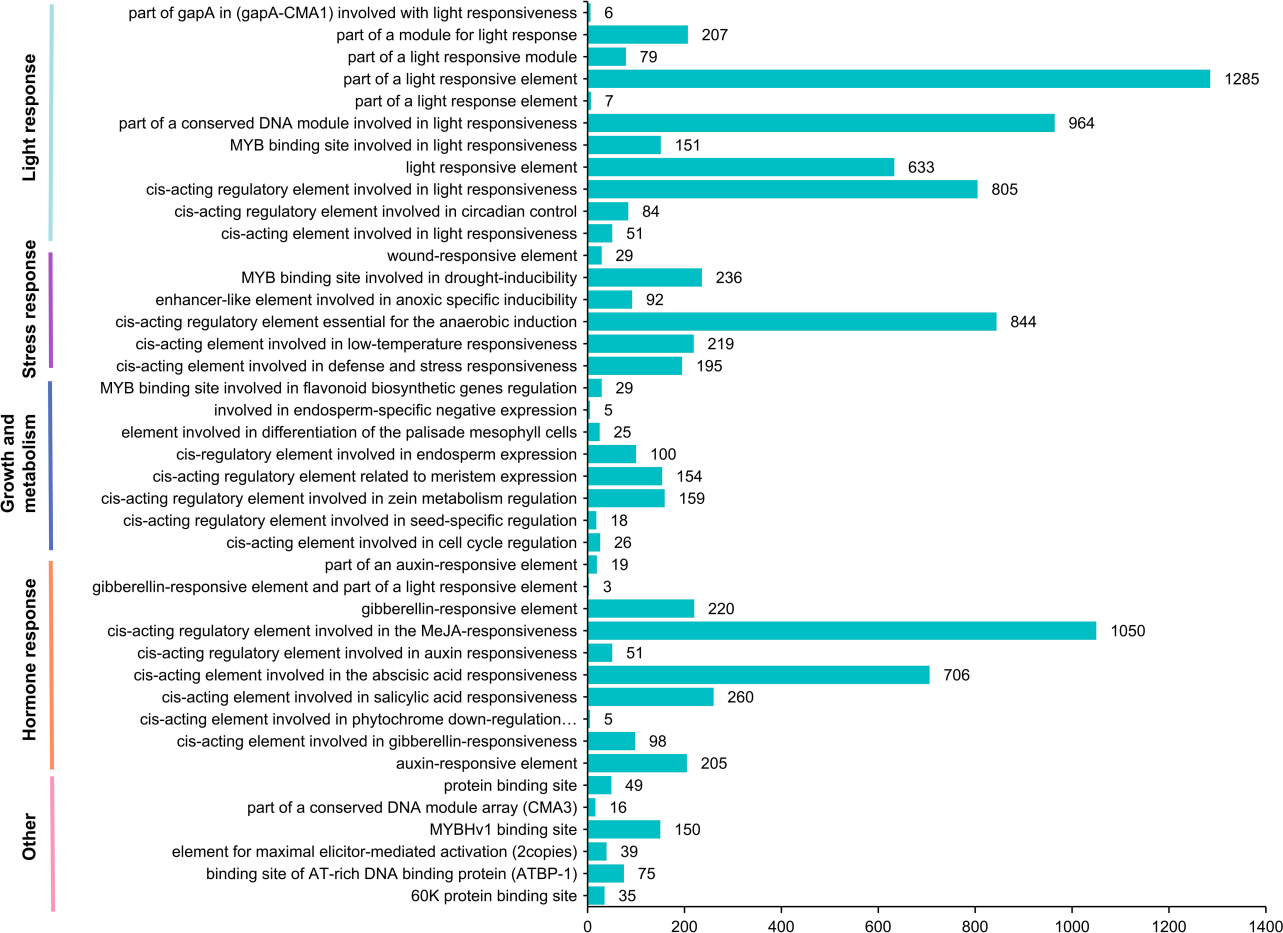
Cis-element analysis of the *LRR-RLK* genes in *A. chinensis*.

Cis-acting elements in the promoter region play crucial roles in gene transcription and expression, making their analysis essential for understanding gene function. We utilized the PlantCare website to predict cis-acting elements in the upstream promoter regions of *AcLRR-RLK* genes. The identified elements were classified into four primary categories: light response, hormone response, stress response, and growth and metabolism (**Figure 4**). In *A. chinensis*, the most abundant elements were light-responsive elements present in all the upstream sequences of *AcLRR-RLK* genes, with G-box and Box 4 elements as the primary conserved motifs. The second most prevalent category was hormone-responsive elements, particularly those related to methyl jasmonate (MeJA) and abscisic acid (ABA) responsiveness, which were abundant in the *AcLRR-RLK* genes. Among the stress-responsive cis-acting elements, those related to anaerobic induction, low-temperature stress, drought stress, and defense against biotic stress were prominent and occupied a significant portion of the upstream promoter regions of *AcLRR-RLK* genes. Finally, elements associated with plant growth and metabolism, relatively fewer in number in *AcLRR-RLK* genes, included conserved motifs such as O2-site, CAT-box, and GCN4_motif.

Further analysis of the distribution of different types of cis-regulatory elements carried by different subfamilies (**Supplementary Figure S3**) revealed that all subfamilies contained many light-responsive elements, with group XIIb being the subfamily that included the most light-responsive element-related elements. Group XIIIb contains the most MeJA (methyl jasmonate) response-related cis-regulatory elements, followed by group IV. Additionally, VIIb is the only subfamily that does not contain MeJA response-related cis-regulatory elements. Xc is the only subfamily that lacks abscisic acid response-related cis-regulatory elements. These results suggest that all subfamilies of *AcLRR-RLK* genes play a role in the light response, but the functions of different subfamilies in other biological processes may differ.

### 3.6 Gene chromosomal location, duplications, and collinearity

The chromosome localization results indicated that *AcLRR-RLK* genes are distributed across 29 chromosomes (**Supplementary Figure S4**). For HapA (**Supplementary Figure S4a**), the greatest number of genes was observed on Chr03, followed by Chr11 and Chr16, whereas Chr07 had the fewest *AcLRR-RLK* genes. In HapP (**Supplementary Figure S4b**), Chr11 had the most genes, closely followed by Chr03 and Chr08, and Chr07 again had the least number of *AcLRR-RLK* genes. Notably, HapP had a significantly greater number of genes on Chr10 than did HapA. HapP also had more *AcLRR-RLK* genes than HapA did on Chr04, Chr08, Chr11, Chr17, Chr20, Chr21, Chr23, and Chr26. Conversely, compared with HapP, HapA presented more genes on Chr05, Chr13, Chr15, Chr19, Chr24 and Chr28. *AcLRR-RLK* genes belonging to the same subfamily were not localized in a few specific chromosomal regions but were instead distributed across multiple chromosomes. Most *AcLRR-RLK* genes were located toward the chromosomal ends (subtelomeric regions), with relatively similar distribution patterns observed in both haplotypes.

**Figure 5:**
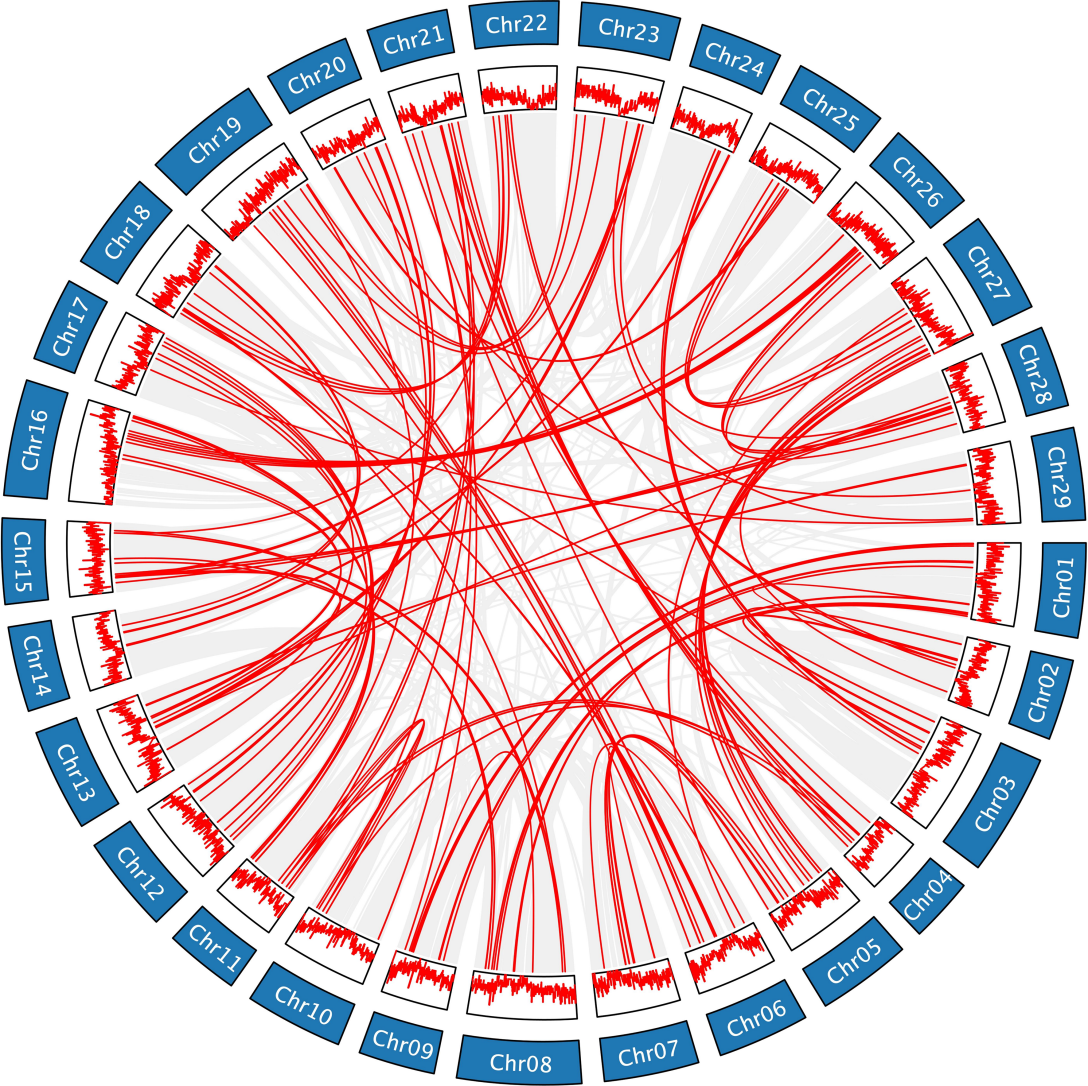
Genomic collinearity of the *AcLRR-RLK* family in *A. chinensis* HapA. Distribution and collinearity of the *AcLRR-RLK* family in the genome. The ring in the middle represents the gene density of each chromosome. The gray background lines represent a collinear background, and the red lines indicate a collinear relationship between *AcLRR-RLK* members.

Gene duplication is one of the primary drivers of genome and genetic system evolution (Moore and Purugganan, 2003). There are three main types of gene duplication, including whole genome duplication, tandem duplication, and segmental duplication, all of which can give rise to numerous gene families (Qiao et al., 2019). According to the JCVI collinearity analysis (**Supplementary Table S3**; **Figure 5**; **Supplementary Figure S5**), the gene duplications of *AcLRR-RLK* genes were all segmental duplications. In HapA and HapP, 135 and 136 pairs of duplicated genes are distributed across different chromosomes, respectively, and these gene pairs are predominantly found in regions with relatively high gene density. The duplicated gene pairs in HapA and HapP included 222 and 225 *AcLRR-RLK* genes, respectively, accounting for more than half of the Hongyang *AcLRR-RLK* genes. Analysis of gene collinearity between the two haplotypes revealed that most *AcLRR-RLK* genes are collinear, with some being haplotype-specific *AcLRR-RLK* genes. HapA and HapP contain 38 and 27 specific *AcLRR-RLK* genes, respectively (**Supplementary Table S1**; **Supplementary Figure S4**), and these specific genes are distributed across multiple chromosomes (**Supplementary Figure S4**). From the distribution of *AcLRR-RLK* genes on chromosomes, some haplotype-specific *AcLRR-RLK* genes are clustered within specific chromosomal regions. In HapA, the specific *AcLRR-RLK* clusters are located on chromosomes Chr08, Chr10, Chr13, Chr16, Chr19 and Chr28. However, the specific *AcLRR-RLK* genes in HapP are more sparsely distributed. These findings suggest that the genes of the *AcLRR-RLK* family are generated through gene duplication, with segmental duplication playing a major role in the expansion of the *AcLRR-RLK* gene family. Additionally, some haplotype-specific *AcLRR-RLK* genes are present between the two haplotypes.

### 3.7 *AcLRR-RLK* gene expression in different tissues

We analyzed the expression patterns of different groups of *AcLRR-RLK* genes in various plant tissues using transcriptome data (**Supplementary Figure S6**; **Supplementary Table S5**). The expression levels of the TPM were measured. We observed that group IV genes were not expressed in flowers, whereas genes from the other groups were expressed in all the tissues. Groups I, II, III, IX, V, VIIb, VIII-2, Xa, Xb, XI, XIIa, and XIV broadly expressed in all tissues, suggesting that these groups may function in multiple tissues. Further combined with gene duplication analysis (**Supplementary Table S3**), it was found that 86% of the genes in group III were duplicate gene pairs, and these gene pairs were generally highly expressed in multiple tissues. This result implies that these duplicated gene pairs in III may maintain their original functions and have functional redundancy. Some groups were highly expressed specifically in certain tissues. For example, groups VIIa, VI, VIII-1, and XV showed specific expression in root, stem, leaf, and fruit; XIIb genes were abundant in root, stem, and leaf; and group IV genes were prevalent in stems. Group Xc is highly expressed in flower, stem, leaf, and fruit, and the promoter region of its genes carry MeJA-responsive elements (**Supplementary Table S2**). MeJA has been reported to be closely associated with floral organ development and opening in a variety of plants, including *Arabidopsis thaliana* and *Brassica napus* L. (Heil et al., 2001; Pak et al., 2017; Wang et al., 2024). Therefore, the Xc subpopulation may coordinate floral development through the MeJA signaling pathway. These findings indicate that most groups have broad functions across all tissues, whereas some may have undergone functional differentiation, acting in several specific tissues or through specific signaling pathways. Furthermore, we found that all the genes were highly expressed in stems, implying that the growth and development of stems require the synergistic action of all the *AcLRR-RLK* subfamilies.

From the genetic level, 30 (79%) HapA-specific genes and 14 (52%) HapP-specific genes were expressed in different tissues without significant tissue specificity (**Supplementary Table S5**). A greater proportion of HapA-specific genes exhibited expression across multiple tissues, suggesting broader involvement in tissue development.

### 3.8 Responses of *AcLRR-RLK* genes under *Psa* infection in *A. chinensis*

*Pseudomonas syringae* pv. *actinidiae* (*Psa*) is the pathogenic bacterium responsible for causing bacterial canker in Mihoutao (Cameron and Sarojini, 2014). The key virulence factor of *Psa* is the protein secretion system involving the type III secretion system (T3SS). This secretion system secretes various toxic effectors capable of disrupting the immune defense responses of the plant, enabling the pathogen to rapidly adapt to the host environment (Jayaraman et al., 2023; Colombi et al., 2024). To investigate the expression patterns of *AcLRR-RLK* in response to *Psa*, we analyzed *AcLRR-RLK* expression levels during *Psa* infection using transcriptome data from phloem tissues collected at different time points after infection. Differential expression gene analysis comparing various time points after pathogen infection with the 0 hour after inoculation (hai) sample revealed that significantly more *AcLRR-RLK* genes were downregulated than upregulated at 12 hai (**Supplementary Figure S7a**). These findings suggest that the early stages of pathogen infection may suppress the expression of certain defense-related *AcLRR-RLK* genes. However, at 24 hai, a notable increase in the number of upregulated genes was observed (**Supplementary Figure S7b**), indicating that plants may start activating *AcLRR-RLK* genes related to disease resistance or the emergency response at this time. As the infection progressed (**Supplementary Figures S7c, d**), the number of upregulated *AcLRR-RLK* genes gradually increased. This implies that during early *Psa* infection, the expression of some *AcLRR-RLK* genes is suppressed, but as infection persists, the defense response of Mihoutao is activated, and an increasing number of *AcLRR-RLK* genes begin to respond to pathogen infection.

**Figure 6:**
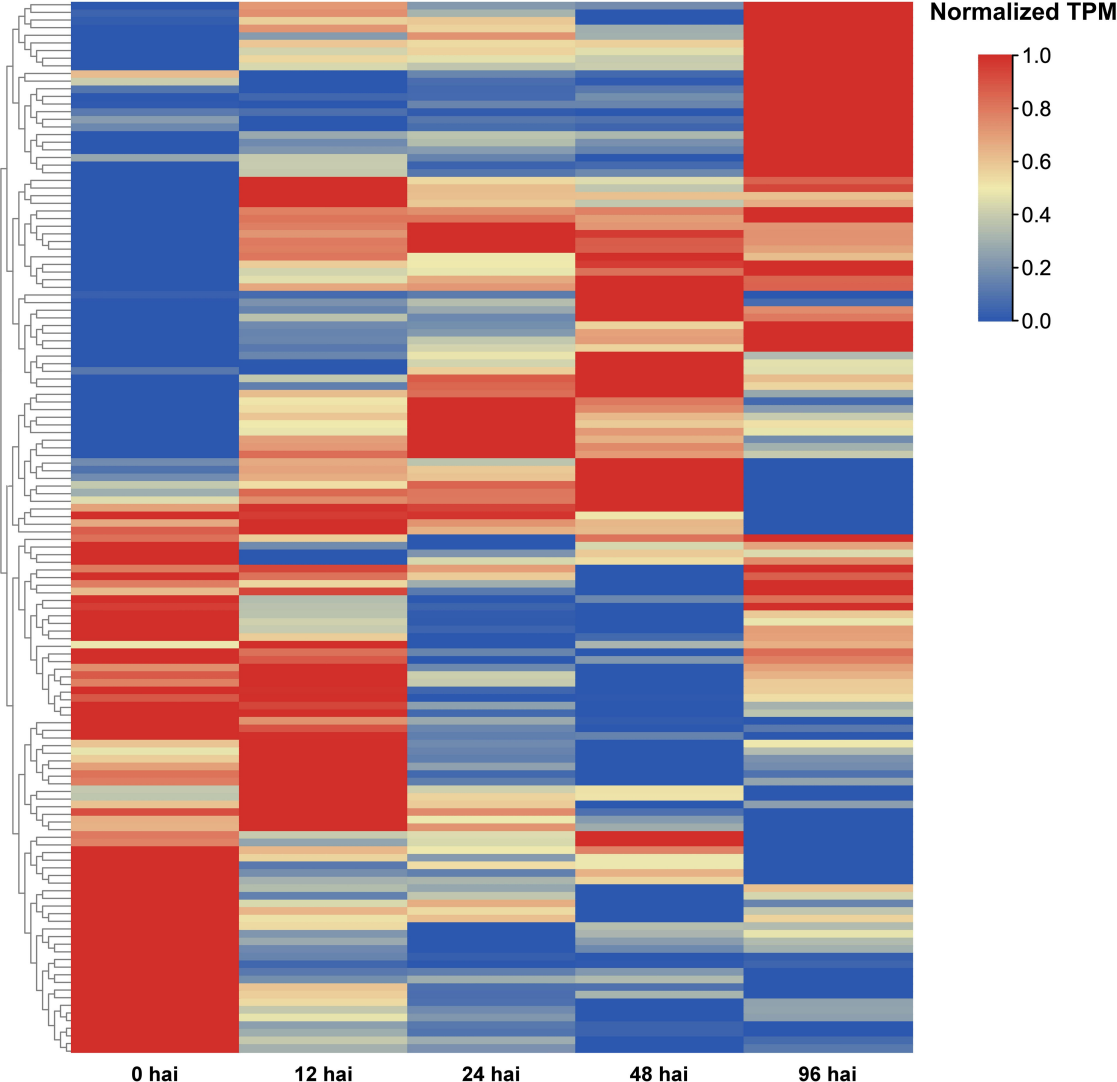
*AcLRR-RLK* genes were significantly different expressed at different time points after *Psa* infection compared with 0 hai infection in *Actinidia chinensis*.

Heatmaps were generated to illustrate the differential expression of *AcLRR-RLK* genes at various time points (12 hai, 24 hai, 48 hai, and 96 hai) after *Psa* infection (**Figure 6**; **Supplementary Table S4**). Compared with 0 hai, the expression levels of certain genes significantly increased during the early infection stage (12 hai), indicating that the plants rapidly initiated a preliminary immune response following pathogen invasion. As the infection progressed (24 hai), the expression levels of these genes further increased, although the expression of some initially highly expressed genes significantly decreased compared with that in 0 hai. This suggests that while the immune response of the plant gradually strengthened, the immune response may have simultaneously suppressed genes related to growth and metabolic activities. At 48 hai, the changes in gene expression peaked, with some defense-related genes that began being expressed in the early and middle stages potentially reaching their maximum expression levels, whereas some genes whose expression was downregulated early continued to be downregulated or maintained at low expression levels. During this phase, the plant significantly adjusts gene expression to combat pathogen invasion, likely in an effort to inhibit pathogen spread or repair damaged tissues. At 96 hai, the changes in gene expression stabilized, indicating that the plant may have adapted to the presence of the pathogen and entered a new physiological equilibrium. Of these *LRR-RLK* genes that responded to *Psa* infection, 12 were HapA-specific genes, and these were predominantly delayed responses (e.g., Achv4a10g015387.t1 peaked at 96 hai), which may be related to hormonal signaling regulation. Seven were HapP-specific genes, which tended to be rapidly activated (e.g., Achv4p29GSAman00020.t1 peaks at 24 hai).

Forty-eight *AcLRR-RLK* genes showed up-regulation at four time points, 12 hai, 24 hai, 48 hai and 96 hai, after *Psa* infection, suggesting that they are continuously activated throughout the pathogen invasion (**Figure 6**; **Supplementary Table S4**). These 48 *AcLRR-RLK* genes belong to 14 subfamilies, including I, II, III, IV, V, VI, VIIa, VIII-1, VIII-2, Xb, XI, XIIa, XIIb and XV. Among these, subgroup XIIa contained the greatest number of genes, with 10 *AcLRR-RLK* genes, followed by the XIIb subfamily, which included 8 *AcLRR-RLK* genes. Interestingly, we also found that two genes lacking the atypical SKG6 domain, Achv4a22g033382.t1 and Achv4a18g026849.t1, reached peak expression at 96 hours and 48 hours after *Psa* infection, respectively (**Supplementary Table S6**). However, three genes containing the atypical SKG6 domain (Achv4a29g045379.t1, Achv4p29GSAman00020.t1, and Achv4a29g045380.t1) showed peak expression as early as 24 hours post-infection. The SKG6 domain is a membrane-associated module exhibiting polarized intracellular localization and includes a highly conserved transmembrane α-helix (Tomishige et al., 2005). This domain may facilitate membrane targeting (Lingwood and Simons, 2010), potentially enabling *LRR-RLK* receptors to be directed to specific membrane microdomains and to sense external stress more rapidly. This mechanism could explain the early induction of SKG6-containing *LRR-RLK* genes during the initial stage of *Psa* infection. Expression analysis suggests these 48 *AcLRR-RLK* genes are key players in basal defense against *Psa* infection, as they were significantly and consistently upregulated across all time points. Subgroup XII, in particular, plays a critical role in defending against pathogen invasion. Combined with gene duplication analysis, we found (**Supplementary Table S3**) that there were 10 duplicated gene pairs in XII, all of which showed significant differences in expression response at different time points of *Psa* infection, suggesting that they may have undergone functional differentiation in pathogen recognition and defense initiation. This result is the same as those of previously reported studies (Dufayard et al., 2017), further confirming the importance of subgroup XII in plant immune responses. These XII subfamily genes are homologous to known functional genes such as *Arabidopsis FLS2* (Chinchilla et al., 2006), *EFR* (Zipfel et al., 2006), and rice *Xa21* (Sun and Wang, 2011), and may serve as potential candidate genes for *Psa* resistance. In addition, we found that loss/gain of atypical domains may affect the response of *LRR-RLK* genes to *Psa*.

To further validate their immune functions, we performed Gene Ontology (GO) enrichment analysis on the 48 core *Psa*-responsive genes (**Supplementary Figure S8**). The results showed that these genes were significantly enriched in several plant immunity-related processes, including cell surface receptor kinase signaling pathways, pathogen recognition, hormone stimulus response, and callose deposition. In the MF category, the genes were enriched in molecular functions such as “kinase activity” and “receptor activity”. These enriched terms are highly consistent with the canonical pattern-triggered immunity (PTI) response in plants. Previous studies have shown that *LRR-RLK* genes are located at the plasma membrane, where they detect pathogen-associated molecular patterns (PAMPs) and activate PTI signaling (Malinovsky et al., 2014; DeFalco and Zipfel, 2021); this is followed by the activation of intracellular signaling pathways such as MAPK cascades, and the deposition of callose and enhancement of lignification in the cell wall, thereby increasing resistance to pathogens (Lloyd et al., 2014; Malinovsky et al., 2014). Therefore, these core *AcLRR-RLK* genes likely mediate defense against *Psa* through a typical “PRR recognition-kinase signaling-cell wall reinforcement” mechanism.

### 3.9 qRT–PCR validation of *AcLRR-RLK* genes expression under *Psa* stress

**Figure 7:**
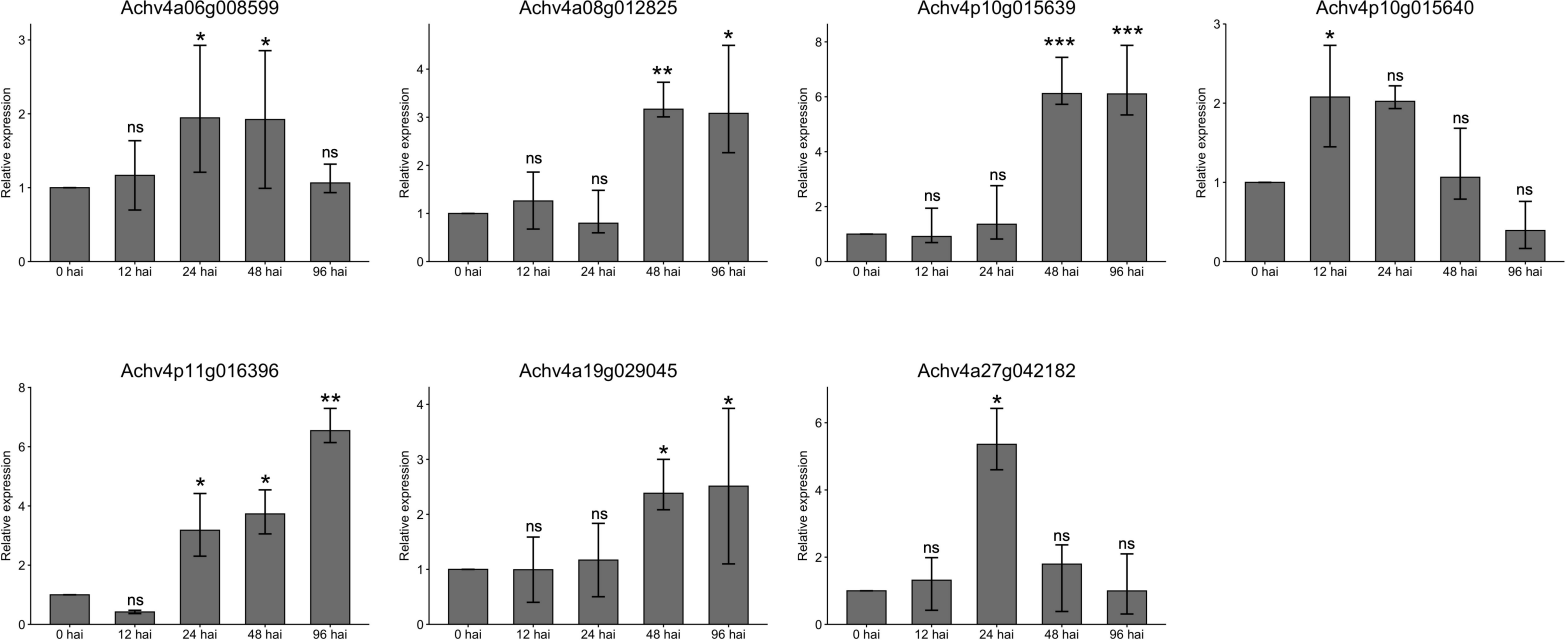
Gene expression of parts of the *AcLRR-RLK* gene family in *Actinidia chinensis* at different time points after *Psa* infection. Seven *AcLRR-RLK* members were detected by qRT-PCR at no inoculation and after infection for 0, 12, 24, 48 and 96 h. Different symbols indicate significance of differences (ns>0.05, *p≤0.05, **p≤0.01, ***p≤0.001), as determined by one-way ANOVA with SPSS single-factor tests..

To validate the transcriptome results, we selected seven representative core *AcLRR-RLK* genes from the XII subfamily responsive to *Psa* based on the phylogenetic tree (**Figure 1**) and conducted qRT-PCR assays at 0, 12, 24, 48, and 96 hours after inoculation (hai). The expression trends of the *AcLRR-RLK* genes after *Psa* infection aligned with the transcriptomic data (**Figure 7**). Specifically, compared to baseline (0 hai), the expression level of *Achv4p10g015640* reached a peak at 12 hai *Psa* infection and then subsequently decreased over time. In contrast, the expression levels of *Achv4a08g012825*, *Achv4p10g015639*, *Achv4p11g016396*, and *Achv4a19g029045* gradually increased during the infection period. Additionally, *Achv4a06g008599* expression was elevated at 24 and 48 hai, followed by a significant decrease at 96 hai. *Achv4a27g042182* exhibited an initial increase at 24 hai and then gradually declined over subsequent time points.

## 4 Discussion

The *LRR-RLK* gene family, a major class of cell surface receptors, regulates key biological processes including plant growth and development, hormone signaling, and defense responses. In this study, we systematically identified and analyzed the *LRR-RLK* gene family in *A. chinensis* cv. Hongyang on the basis of typical domains. All the annotated genes contained at least one LRR domain, a TM domain, and a kinase domain. We identified 336 and 351 *LRR-RLK* genes in the two haplotypes of *A. chinensis* (HapA and HapP), respectively, and manually corrected the gene structures to ensure accurate annotation. These genes were classified into 21 subfamilies, which is consistent with the classification of *Arabidopsis thaliana* and *Oryza sativa* (Shiu et al., 2004; Sun and Wang, 2011). The kinase domains of the *AcLRR-RLK* genes were highly conserved, whereas the LRR domains were highly diverse. Structurally, genes within the same subfamily shared similar structures, whereas significant differences were observed among subfamilies. This structural specificity and diversity support the functional differentiation of the *LRR-RLK* genes (Man et al., 2020). Through the analysis of gene expression patterns, this study further revealed the importance of the *AcLRR-RLK* gene family in the growth, development, and disease defense of Mihoutao. Most genes were expressed in various tissues, with some genes showing specific high expression in flowers, stems, and fruits, suggesting their potential association with tissue-specific functions in Mihoutao. Additionally, through the prediction and analysis of cis-acting elements, the promoter regions of *AcLRR-RLK* genes were found to be rich in cis-acting elements related to the light response, hormone regulation (such as ABA and MeJA), abiotic stress (such as drought and low temperature), and disease response, further emphasizing the indispensable role of this gene family in multiple biological processes.

The *LRR-RLK* gene family has been identified in various plants in recent years. In *Phaseolus vulgaris* L., 230 *LRR-RLK* genes distributed across 15 subfamilies have been identified (da Silva Dambroz et al., 2023). In *Arachis hypogaea* L., 495 *LRR-RLK* genes have been detected (Wang et al., 2024). Similarly, *Saccharum* sp*ontaneum* harbors 288 *LRR-RLK* genes (Ding et al., 2024). The model crop *A. thaliana* has a total of 240 genes belonging to 21 subfamilies of *LRR-RLK* genes. Compared with the findings in *Arabidopsis*, the *LRR-RLK* genes in Mihoutao are also categorized into 21 subfamilies, with group III being the largest, indicating their potentially significant evolutionary role in plant genomes (Shiu et al., 2004; Sun and Wang, 2011). The highly conserved kinase domain and diverse LRR domains of the *LRR-RLK* genes observed in this study further reinforce their role as key regions for functional differentiation, similar to previous reports (Dufayard et al., 2017). Compared with *Phaseolus vulgaris* L., *A. chinensis* harbors a greater proportion of *LRR-RLK* genes with atypical domains (accounting for 39% of all *AcLRR-RLK* genes), which are distributed across 19 subfamilies, indicating a broader and more diverse structural complexity. Among these, the most prevalent atypical domains in *AcLRR-RLK* genes are TM_ErbB1 and Syndecan. *AcLRR-RLK* genes carrying these domains are distributed primarily in groups I, II, III, IV, IX, V, VIII-2 and XIIa, suggesting that these subfamilies may play roles in growth and development. This hypothesis is further supported by tissue-specific expression analysis of *AcLRR-RLK* genes, which highlights their potential involvement in developmental processes. For example, the XV subgroups were highly expressed in fruits (**Supplementary Figure S5**), and the promoter regions of these genes carry hormone-associated elements in response to growth hormone, gibberellin, abscisic acid, and other hormones, which may be involved in fruit growth and ripening. Functional validation of these genes would clarify their roles, providing insights for improving fruit development and ripening.

In *A. chinensis*, a significant temporal expression pattern of *AcLRR-RLK* was observed during *Psa* infection, which is consistent with the expression pattern of *LRR-RLK* in *A. thaliana* (Dievart and Clark, 2004). This finding suggests the existence of a conserved immune response pathway for *LRR-RLK* genes across species. Through the analysis of *LRR-RLK* gene expression patterns after pathogenic bacterial infection, this study revealed the potential role of these genes in disease resistance. Some genes were suppressed early in the infection process. During the early stages of *Psa* infection (12 hai), most *AcLRR-RLK* genes were downregulated, possibly due to the pathogen’s secretion of effector molecules that inhibit the plant’s PTI response (Jayaraman et al., 2023; Colombi et al., 2024). These *AcLRR-RLK* genes, which are suppressed during the early stages of infection, may be “virulence targets” for pathogen action, host susceptibility factors (S genes) that are utilized by the pathogen to promote infection. As the infection progressed, more *LRR-RLK* genes were activated, indicating that the plant may gradually initiate mechanisms related to disease defense or the emergency response. Forty-eight *AcLRR-RLK* genes were consistently up-regulated for expression at four time points after *Psa* infection. The highest concentration of genes was found in XII subgroup, with 18 of these 48 genes belonging to XII. These subgroup XII genes are homologous to several known functional genes such as *FLS2*, *EFR*, and *Xa21* (Chinchilla et al., 2006; Zipfel et al., 2006; Sun and Wang, 2011). Functional validation of these genes involved in *Psa* resistance may provide candidate targets for breeding *Actinidiaceae* family crops with enhanced resistance.

The function of *LRR-RLK* genes in disease defense also have been reported in other fruit crops. Among *LRR-RLK* genes identified in citrus, the expansion and tandem duplication of subgroup XII genes are significantly upregulated upon *Xanthomonas citri* infection (Magalhães et al., 2016). In grapevine, 42.5% of *LRR-RLK* genes belong to expanded clades, and multiple genes from the XII, XI, and II subgroups-such as *VvSERK3*, *VaHAESA*, and *VvFLS2*—are involved in PAMP recognition (Tang et al., 2010; Trdá et al., 2014; Liu et al., 2018; Li et al., 2021). In apple, *LRR-RLK* genes from subgroups II, XI, and XII (e.g., *MdBAK1*, *LRPKm1*, and *MdFLS2-1*) have also been shown to participate in immune responses against various pathogens (Komjanc et al., 1999; Liang et al., 2022; Liu et al., 2022). These studies indicate that subgroups II, XI, and XII are commonly involved in pathogen recognition and immune signaling across multiple fruit species. We found that *A. chinensis* also harbors a relatively large number of *LRR-RLK* genes in subgroups II, XI, and XII, with many genes significantly induced during *Psa* infection. In particular, several subgroup XII genes were continuously upregulated at various stages of infection, suggesting their central role in pathogen detection and defense signal initiation. In contrast (**Supplementary Figure S9**), citrus contains fewer genes in subgroups II and XI, while grapevine has a notably lower number of subgroup XII genes. These results suggest that the expansion or retention of these key subgroups varies among fruit species, reflecting both functional conservation and species-specific evolutionary divergence.

Integration of transcriptomic, proteomic and metabolomic data can analyze gene functions and regulatory networks from multiple dimensions, which can significantly enhance the understanding of plant disease resistance mechanisms. Transcriptomic data (e.g., TPM values) reveal gene expression levels, but mRNA abundance may not correspond to protein abundance and function. We found that some genes of subgroup XII were significantly up-regulated after *Psa* infection, and if we further confirm the changes in protein abundance and kinase activity by combining tandem mass tag (TMT) quantitative proteomic and phosphoproteomic data, and observe the accumulation of related disease resistance metabolites by combining the data of targeted and non-targeted metabolomes, it would be helpful to construct the pathway of “*LRR-RLK*→kinase cascade→metabolic defense”, and to reveal the role of its in the immune signal transduction. This multi-omics strategy can screen the key genes, proteins and metabolites in response to *Psa* stress at multiple levels, and clarify whether the candidate *AcLRR-RLK* genes can be converted into functional proteins, avoiding the bias caused by single-omics, and providing more precise targets for downstream functional validation and breeding efforts. In the future, we will combine proteomic and metabolomic data to deeply analyze the function of the *AcLRR-RLK* gene, construct a regulatory network from signal perception to phenotypic output, and promote the molecular design of disease-resistant varieties.

The expansion of the *AcLRR-RLK* gene family is achieved primarily through segmental genome duplication, a pattern of gene duplication identical to that of *Actinidia eriantha* ‘Huate’ (diploid) (Cao et al., 2024), which may represent one of the significant driving forces in the evolution of the diploid Mihoutao genome (Moore and Purugganan, 2003; Qiao et al., 2019). Whereas in *Actinidia arguta* (tetraploid), the mode of replication of resistance genes is mainly whole genome duplication (WGD) (Kataoka et al., 2010), in *Actinidia chinensis* var. *deliciosa* (hexaploid), a combination of WGD and segmental duplication plays a dominant role (Wang et al., 2018; Zhang et al., 2023). Additionally, 38 and 27 haplotype-specific *LRR-RLK* genes were identified in HapA and HapP, respectively. Transcriptome analysis revealed that HapA-specific *LRR-RLK* genes were mainly involved in plant development-related processes, reflecting their adaptive evolution in bryophyte morphogenesis. In contrast, HapP-specific genes are more inclined to mediate rapid stress response and enhance defense against pathogens, reflecting their functional bias and adaptive divergence under long-term pathogen selection pressure.

Our study analyzed the *LRR-RLK* gene family in *A. chinensis* at the genomic haplotype and transcriptome levels, enriching the understanding of this gene family. We demonstrated the haplotype specificity of the *LRR-RLK* gene family and identified *LRR-RLK* genes that respond dynamically to pathogenic infection. These findings provide a new perspective for plant gene family research. Some haplotype-specific *LRR-RLK* genes with tissue-specific or *Psa*-induced expression may serve as molecular markers for Mihoutao strain improvement and *Psa* resistance breeding. For example, the HapP-specific gene Achv4p29GSAman00020.t1 responded rapidly after *Psa* infection (peaked at 24 hai, **Supplementary Table S4**) and may activate defense responses by phosphorylating downstream immune signaling proteins. The HapA-specific gene Achv4a16g024156.t1 had the highest level of expression in stems, which may regulate mechanical support of bryophytes. These genes may be candidates for the development of molecular markers for resistance breeding and strain improvement. In addition, *LRR-RLK* genes that are specifically expressed in tissues and carry elements related to plant growth and development (e.g., VIIb and XV groups) and core *LRR-RLK* genes that respond to *Psa* infection can also be used as molecular designed breeding targets for Mihoutao growth and disease resistance. Although we have not conducted in-depth functional studies on these key genes, the multiple disease-response- and growth- related genes identified in this study offer candidate targets for functional validation and molecular breeding of Mihoutao. Our study constructed a comprehensive *AcLRR-RLK* gene resource database covering 394 manually corrected genes and their expression profiles in different tissues and *Psa* infection conditions. This database also lays a solid foundation for subsequent functional studies and breeding utilization of disease resistance genes.

## 5 Conclusions

This study conducted annotation, structural refinement, and systematic analysis of the *LRR-RLK* genes in the genome of *Actinidia chinensis* cv. Hongyang, revealing 394 *AcLRR-RLK* genes that were classified into 21 subfamilies with homologous *LRR-RLK* genes in *Arabidopsis thaliana*. In *A. chinensis*, group XIIa and III had the largest number of genes, whereas group Xc had the fewest genes. Analysis of gene structure and conserved motifs revealed high diversity in the LRR domain, whereas the kinase domain was relatively conserved. Cis-acting element analysis revealed that *AcLRR-RLK* genes are involved in various important biological processes in plants, demonstrating the functional importance of this family in *A. chinensis*. Our study revealed that gene expansion in the *AcLRR-RLK* gene family is due mainly to gene replication, and we identified two haplotype-specific *AcLRR-RLK* genes. Transcriptome data analysis revealed that most *AcLRR-RLK* subfamilies play roles in five tissues: root, stem, leaf, flower and fruit. Several subfamilies, such as VIIa, VI, VIII-1, Xc and XV, function in several specific tissues. In addition, analysis of the expression of *AcLRR-RLK* genes during *Psa* infection revealed 48 core genes related to the response to this pathogen, among which subgroup XII may play a key role. This study provides valuable insights into the characteristics of the *LRR-RLK* family in *A. chinenses*, differences between haplotypes, and responses to biological stress, laying a molecular foundation for the utilization of this gene family and the breeding of varieties resistant to *Psa*.

## Data Availability

The original contributions presented in the study are included in the article/**Supplementary Material**. Further inquiries can be directed to the corresponding author.

## Supplementary material

Supplementary File 1 Phylogenetic tree, conserved motifs, and gene structure of *AcLRR-RLK* genes in Hongyang. The phylogenetic tree of *AcLRR-RLK* genes was constructed on the basis of kinase sequences. The arrangement of conserved motifs in each *AcLRR-RLK* was visualized using conserved motif information, with colored boxes representing motifs 1-15. The gene structures of *AcLRR-RLK* genes were visualized for the cds region, with the green boxes representing the cds areas.

Supplementary Figure 1 Domain architectures of the *AcLRR-RLK* subfamilies. **(a)** Domain architecture of LRR-I subfamily. **(b)** Domain architecture of LRR-VIII-2 subfamily. **(c)** Domain architectures of LRR-Xc and LRR-VIIa subfamilies. **(d)** Domain architectures of all remaining subfamilies.

Supplementary Figure 2 Atypical domains carried by *AcLRR-RLK* genes. The vertical axis represents the types of atypical domains, with only those present in two or more genes specifically labeled. “Other” indicates other atypical domains that occur only in one *LRR-RLK*. The horizontal axis represents the number of atypical domains.

Supplementary Figure 3 Analysis of the cis-acting elements of *AcLRR-RLK* proteins in Hongyang. The heatmap displays the types and numbers of cis-acting elements carried within the 21 *AcLRR-RLK* subfamilies. The numbers in the boxes represent the average number of cis-acting elements in each subfamily, and different colors indicate the range of cis-acting elements.

Supplementary Figure 4 Distribution of haplotype-specific *LRR-RLK* genes on chromosomes in *A. chinensis*. **(a)** Locations of HapA *AcLRR-RLK* genes on chromosomes. **(b)** Locations of HapP *AcLRR-RLK* genes on chromosomes. All haplotype-specific *AcLRR-RLK* genes are marked in blue.

Supplementary Figure 5 Genomic collinearity of the *AcLRR-RLK* family in HapP of Hongyang. Distribution and collinearity of the *AcLRR-RLK* family in the genome. The middle ring represents the gene density of each chromosome. The gray background lines depict a collinear background, whereas the red lines indicate a collinear relationship among *AcLRR-RLK* members.

Supplementary Figure 6 Heatmap of the expression of 21 *AcLRR-RLK* subfamilies in different tissues of *A. chinensis*. We defined TPM>1 as genes expressed in tissues. Brown and white indicate the expression levels of genes in the heatmap: the stronger the red color is, the higher the gene expression level, whereas the stronger the white color is, the lower the gene expression level. HF: Hongyang fruit. HFR: Hongyang flower. HG: Hongyang root. HL: Hongyang leaf. HST: Hongyang stem.

Supplementary Figure 7 Volcano plots of *AcLRR-RLK* genes differentially expressed at different time points after *Psa* infection compared with 0 hai in Hongyang. **(a)** Volcano plot of *AcLRR-RLK* genes differentially expressed at 12 hai compared with 0 hai after *Psa* infection. **(b)** Volcano plot of *AcLRR-RLK* genes differentially expressed at 24 hai compared with 0 hai after *Psa* infection. **(c)** Volcano plot of *AcLRR-RLK* genes differentially expressed at 48 hai compared with 0 hai after *Psa* infection. **(d)** Volcano plot of *AcLRR-RLK* genes differentially expressed at 96 hai compared with 0 hai after *Psa* infection. Red dots represent significantly upregulated genes. The blue dots represent significantly downregulated genes. The gray dots represent genes whose expression did not significantly differ.

Supplementary Figure 8 GO enrichment analysis of 48 core *Psa*-responsive *AcLRR-RLK* genes. **(a)** Enriched Biological Process (BP) terms in 48 core *Psa*-responsive *AcLRR-RLK* genes. **(b)** Enriched Molecular Function (MF) terms in 48 core *Psa*-responsive *AcLRR-RLK* genes.

Supplementary Figure 9 Distribution of *LRR-RLK* genes subfamilies in four fruit crops.
