# Supplementary figures and images for: Genome-wide identification and expression analyses of the LRR-RLK gene family in Actinidia chinensis
Source: Front Plant Sci. 2025 Sep 1;16:1577679. doi: 10.3389/fpls.2025.1577679 (PMC12434026; doi:10.3389/fpls.2025.1577679)

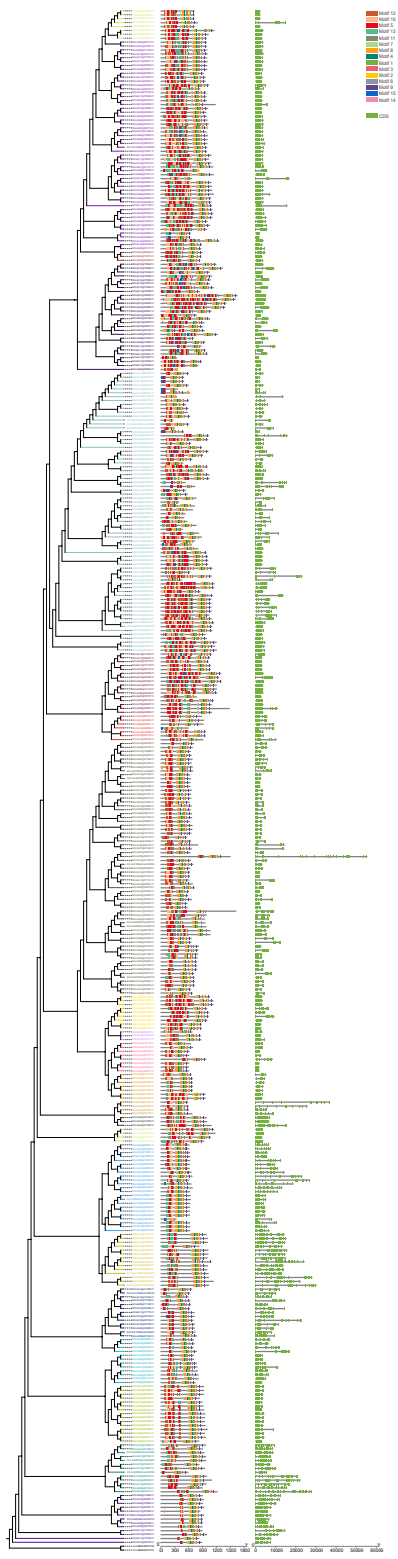

Supplement: Supplementary File 1 — Phylogenetic tree, conserved motifs, and gene structure of AcLRR-RLK genes in Hongyang. The phylogenetic tree of AcLRR-RLK genes was constructed on the basis of kinase sequences. The arrangement of conserved motifs in each AcLRR-RLK was visualized using conserved motif information, with colored boxes representing motifs 1-15. The gene structures of AcLRR-RLK genes were visualized for the cds region, with the green boxes representing the cds areas. [file DataSheet1.pdf]
